# Unnecessary Pain in Dental Operations

**Published:** 1893-07

**Authors:** James A. Reilly


ARTICLE III.

### UNNECESSARY PAIN IN DENTAL OPERATIONS.

BY DR. JAMES A. REILLY.

One forenoon during my senior year at the Harvard Dental School, and while in charge of the dental department of the Bennett Street Dispensary, among the numerous patients was a lad of twelve or thirteen years He went through the usual preliminaries required to have an inferior bicuspid tooth extracted. The operator mechanically picked up his mirror and pliers to examine the tooth, or what remained of it, and almost simultaneously with their introduction into the boy's mouth there was a terrific scream and a plunge that almost carried him through the window. An attempt at extraction by a street dentist had resulted in the removal of the crown, leaving the entire coronal portion of the pulp standing unprotected. The dentist simply plunged his pliers into the mass of living tissue. Was not that an abuse utterly reprehensible on his part? I think it was, and so would you, I believe, had you been the sufferer. Yet we are doing just such things every day in one form or another.

That "familiarity breeds contempt" is nowhere more noticeable than in the use of dental instruments and appliances. Not long since, a gentleman somewhat prominent in dental organizations, told me he had not a dozen excavators in his possession; that he excavated all his cavities with the aid of the dental engine, and wished to wager me that I could not find a cavity in a tooth that he could not reach and prepare as well, if not better, with the engine than it could be accomplished with hand excavators. On being questioned if his patients did not complain of being hurt, he replied: "Confound the patients! my duty is to protect myself." If this gentleman could but be patient and operator at the same time, I have no doubt he would be easily induced to trade some of his burs for hand excavators. Has he not, to say the least, become to "familiar" with his engine?

A prominent writer says, if it is at all difficult to apply, the rubber-dam should not be used in the cases of the very young, very sensitive, or very nervous patients. How many of us draw the line at these classes? It is not my intention to point out the occasions for its use or to urge on you its abandonment, for I consider it a *sine qua non* to good results in numberless cases But I would like to call your attention to the contempt for your patients' feelings that a "familiarity" with its application breeds.

You are all aware how quickly you jerk your head away if by accident the floss slips too rapidly between your own teeth and burrows itself in your gums. How often the same thing is perpetrated on your patients, and nothing thought of it by you, while you are laying coil after coil of cable on teeth that often do not require ligatures! Frequently, indeed, they serve only to obstruct access to the cavity. With holes of proper size and shape in the dam the employment of ligatures is seldom necessary.

It once fell to my lot to sit in the chair with a clamp on a lower wisdom tooth, compelling me to keep open house during the space of three and one-half hours. My knowledge now teaches me that it was entirely unnecessary, and that the cavity might have been filled, with the aid of napkins, in less time than it took to get the rubber and clamp adjusted, and with infinitely less pain and discomfort.

I once saw a case of regulating that was worthy of the attention of the Society for the Suppression of Cruelty to Children. The teeth were very much displaced, and appliances were adjusted to almost all the teeth simultaneously. Too much force was applied, and too long an interval allowed to elapse before changing, so that when I saw the mouth there was scarcely a tooth in the upper maxilla that could not have been easily removed with the fingers. For articulation the patient could not bring the teeth together without suffering intense pain. And all this under the direction of a reputed skilful operator.

Operations really painful, though to us they seem very trifling, which our patients shrink from, we are prone to ascribe to fear or timidity, when we really are inflicting pain. By the habits of some dentists one would suppose the patient had no rights the dentist is bound to respect. He lolls over and leans on his patient, making of their head or their breast a cushion and support for his arm till the patient is well nigh exhausted. It does not diminish the discomfort any to know that it is sometimes done unconsciously. That much inconvenience and unnecessary pain are caused by our neglect to scrutinize our processes and individual peculiarities, or by failure to keep them before our eyes, is not to be denied. Is not unnecessary pain frequently caused while putting on gold caps, bridges, and collars for crowns, without first applying cocain to the gum margin? Is it not unnecessary pain to continue nibbing at an exposed pulp that had not wholly succumbed to the arsenious taste? To catch the lip beneath the thumb while making it a fulcrum against the teeth is painful, and to wash a cavity with cold instead of tepid water may produce avoidable pain.

How common an experience it is to hear an outcry, or see a twitching of the head and body immediately on using the chip-blower while excavating! It does not take place so much if we use warm air. Yet, do we always use it? Is it not positively abusive to whack away at a tooth for hours with the automatic mallet, when hand-pluggers might be used with so much more comfort, at least during the first part of the filling? Is it not an abuse to inflict quick wedging as ordinarily performed? Is it not an abuse in taking full impressions for artificial dentures, to overflow the plaster from your impression-cup into the throat of your patient, when a smaller quantity would produce a much better result by giving a more accurate impression, because the parts are not so likely to be disturbed by retching and coughing? Is it not abusive for a dentist having a strong, muscular hand, with a heavy touch and a vise-like grip, to rush and hurry through with his work as if he were under the impulse of electricity? Rapid operators hurt more than slow ones. I believe after a fair rate of speed has been attained, any acceleration of it is obtained only at the expense of delicacy of touch and of the patient's nervous system.

It is within the ability of everybody to cultivate a delicacy of manipulation, if they do not naturally possess it, and delicate manipulation is a powerful factor in dispelling the dread so universal in the minds of the people relative to dentistry. As President Elliott said the other day at the meeting in behalf of Harvard's new dental school: "It is the dread of pain which makes people miserable."—*Dental Journal.*

